# Trends in immune-related adverse events for colorectal cancer: A bibliometric analysis

**DOI:** 10.3389/fonc.2022.1024321

**Published:** 2022-10-27

**Authors:** Jin Cui, Ying Xiong, Min Sun, Xinyue Gu, Yuting Liu, Luhui Zhong, Xiaohua Hong, Li Liu

**Affiliations:** Cancer Center, Union Hospital, Tongji Medical College, Huazhong University of Science and Technology, Wuhan, Hubei, China

**Keywords:** colorectal cancer, bibliometric analysis, adverse events, immune, co-occurrence

## Abstract

**Purpose:**

We used bibliometric methods to assess the global scientific output on the IRAEs for colorectal cancer and to explore the current status and trends in the field over the last three decades.

**Methods:**

Studies on immune-related adverse events for colorectal cancer published from 1996 to 2022 were retrieved from the Web of Science. For quantitative and qualitative assessments of publication outputs and author contributions, the R bibliometrix package was used. VOSviewer was used to construct networks based on the co-authorship of countries/institutions/authors, co-citation analysis of journals/references, citation analysis of documents, and co-occurrence of keywords.

**Results:**

A total of 237 relevant articles were included in the final analysis. The number of publications has increased significantly over time. The countries and institutions that contributed most to the field were the USA and the University of Texas MD Anderson Cancer Center. Jefferey Schlom was the most productive author, ranking first in cited authors. The most cited document was Topalian et al. in The New England Journal of Medicine (2012). The journals with the highest number of selected articles and citations were The New England Journal of Medicine and the Journal of Clinical Oncology, respectively. Co-occurrence analysis showed that IRAEs for colorectal cancer were associated with immunotherapy, open-label, chemotherapy, nivolumab, and PD-1. Trend analysis showed that immune checkpoint inhibitors, gut-microbiota, inflammatory-bowel disease, and PD-1has been on the rise in recent years to IRAEs for colorectal cancer.

**Conclusion:**

Our bibliometric analysis showed that studying IRAEs for colorectal cancer is increasingly a hot topic. The focus of the research had evolved from traditional treatment modalities such as targeted therapy to gut microbiota. Inflammatory bowel disease may be a future research hotspot of IRAEs for colorectal cancer.

## Introduction

Worldwide, colorectal cancer ranked third in terms of incidence but second in terms of mortality. In 2020, it was estimated that there would be over 1.9 million new colorectal cancer cases and 935,000 deaths, representing approximately 1% of every ten cancer cases and death (1). In recent years, immunotherapy has achieved significant efficacy in treating solid tumors and has been used as a first-line treatment for patients with certain advanced solid tumors (2–4). Although inhibition of programmed death-1 (PD1) or programmed cell death ligand 1 (PD-L1) therapies have had limited success in treating colorectal cancer, immunotherapy is now being used as second-line treatment for patients with dMMR/MSH-H (approximately 15% of patients with colorectal cancer) (5, 6). Immunotherapy is less toxic than traditional treatments such as chemotherapy and targeted therapies. However, it still has some unique side effects called immune-related adverse events (IRAEs), such as skin, liver, gastrointestinal and pulmonary toxicity (7). IRAEs may reduce the clinical efficacy of immunotherapy, and some serious adverse effects may even be life-threatening. A comprehensive and in-depth understanding of colorectal cancer IRAEs is an essential topic for researchers. Bibliometric analysis is a literature synthesis technique that helps to understand further the research and trends in IRAEs for colorectal cancer. Therefore, a bibliometric study is necessary to analyze the published literature and identify current research hotspots and trends.

Bibliometrics takes the global literature as the object of study. It used statistical and other methods to study the distribution patterns and keywords of the literature to explore current research characteristics and practices (8). Through bibliometric studies, analysis of source journals, citations, co-authorship network analysis, distribution of texts, etc., can help researchers to understand further the relevant fields and research trends (9). Currently, there are no bibliometric studies on IRAEs for colorectal cancer worldwide. Therefore, we conducted a systematic bibliometric analysis of the current literature regarding IAREs for colorectal cancer.

## Materials & methods

### Data source and search strategy

All data were retrieved from the Web of Science (WoS) core collection, including SCI-EXPANDED, on August 1, 2022. WOS is one of the most widely accepted and suitable databases for bibliometric analysis of subsequent scientific publications due to its rigorous evaluation process and the most impactful and credible information it can provide. The data for this study were collected from the core collection of the WOS. Documents were collected using the search formula “(TS=(colorectal cancer)) AND (TS=(adversarial events) or TS=(side effects)) AND (TS=(immune))”. The article type filter was set to journals and conference articles, and the language filter was set to English. Collected articles with full records and cited references were saved as plain text for subsequent analysis. In addition, to get the correct number of citations per document We also cross-matched the number of citations for each document in the Scopus and Google Scholar databases.

### Bibliometric analysis and visualization

The bibliographic information of the selected publications was automatically converted and analyzed using the bibliometrix package (10) in R 4.2.1, including trends in research content, country/region, and author distribution. A network of country/institution/author co-authorship analysis, journal/reference co-citation analysis, citation analysis of literature, and keyword co-occurrence analysis was constructed using VOSviewer software version 1.6.18 (11). Keywords that appeared more than five times were presented in three visualizations of the co-occurrence analysis (network, overlay, and density visualization) to identify significant terms in studies of IRAEs for colorectal cancer.

## Results

### The trends in global publications

**Figure 1:**
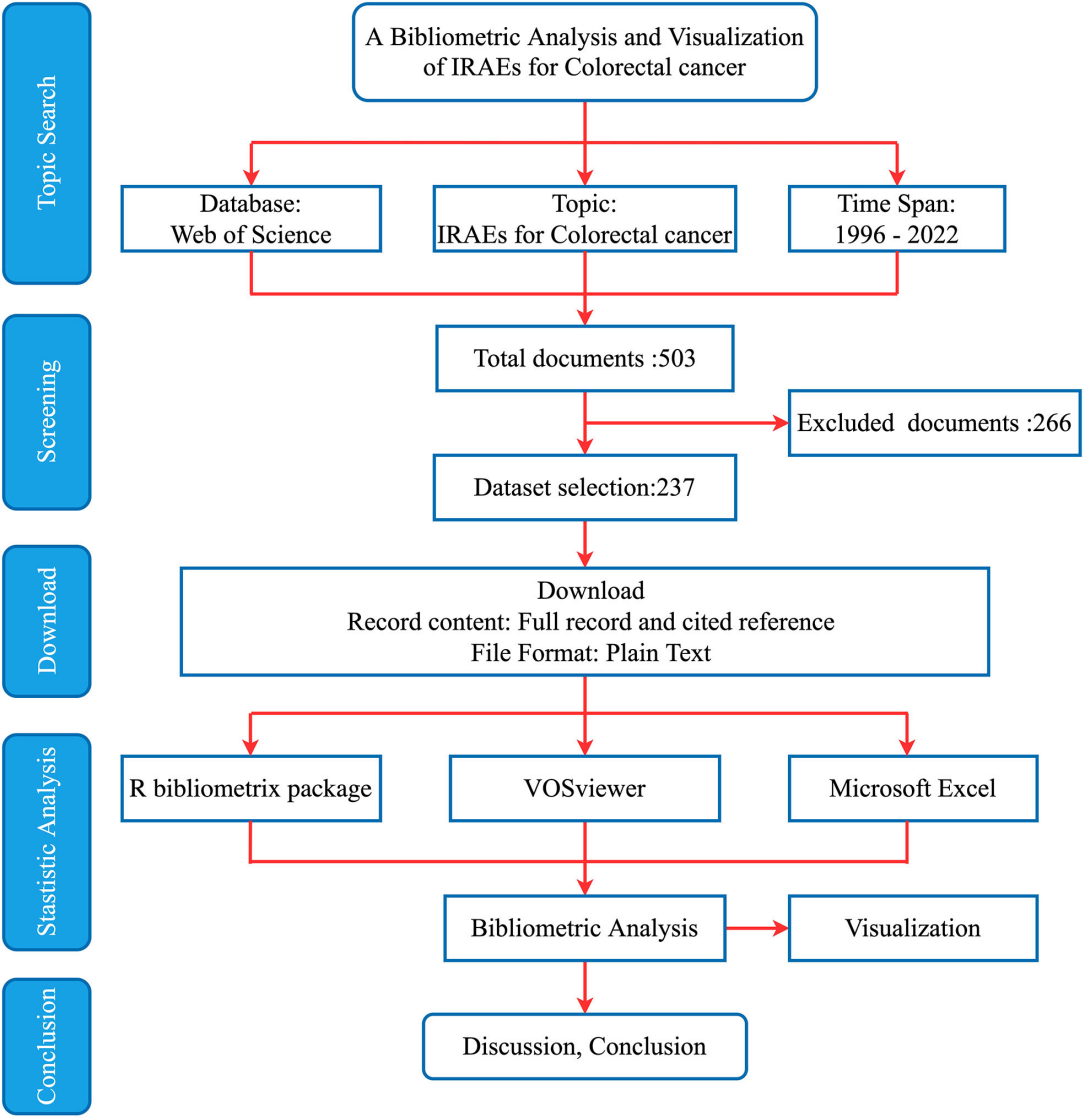
Flowchart of the bibliometric analysis.

**Figure 2:**
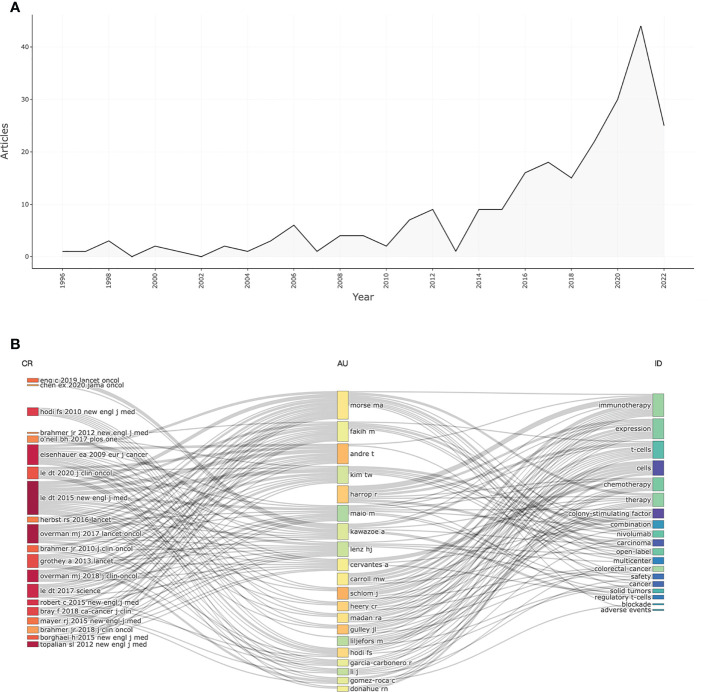
The trends in global publications of the IRAEs for colorectal cancer. **(A)** Trends in literature publication. **(B)** The relationship between references, authors, and keywords.

The flowchart for this bibliometric analysis was shown in **Figure 1**. The leading information of the documents was showed in **Table 1**. A total of 237 articles related to IRAEs for colorectal cancer from 1996 to 2022 were retrieved from the WoS. Global publications in this field showed a strong growth trend from 1996 to 2022 in the number of publications (**Figure 2A**). **Figure 2B** showed the relationship between references, authors, and keywords for inclusion in the literature.

**Table 1:** Main information of included studies.

Description	Results
Timespan	1996:2022
Documents	237
Annual Growth Rate (%)	13.18
Average citations per doc	79.59
References	10960
Keywords Plus (ID)	905
Author's Keywords (DE)	629
Authors	2184
Co-Authors per Doc	10.3
International co-authorships %	24.89

### Distribution of countries and institutions

**Figure 3:**
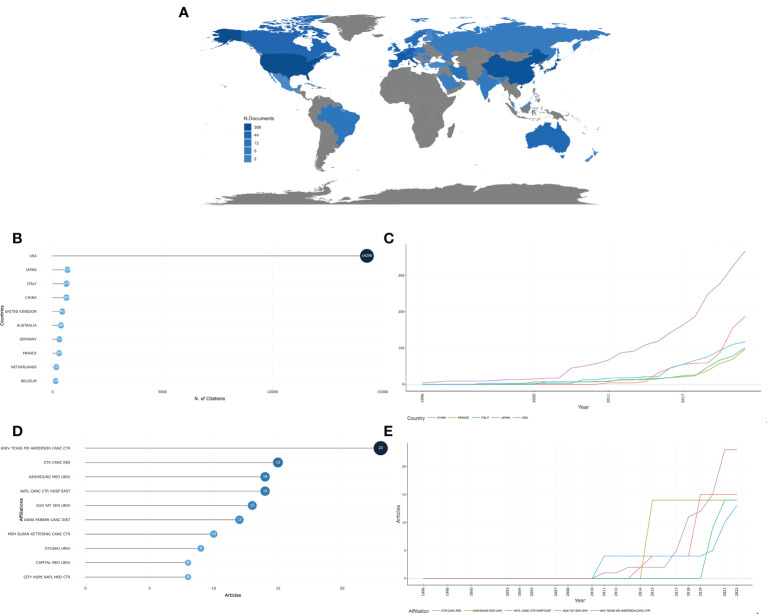
Countries and institutions contributing to IRAEs for colorectal cancer.**(A)** World map showing the distribution of countries in this field. **(B)** Total number of citations of published research by country and region. **(C)** Growth curve of the number of publications per year for the top five contributing countries and regions. **(D)** Distribution of the number of studies published by research institutions. **(E)** Growth curve of the number of publications per year for the top five contributing institutions.

Global contributions to research on the IRAEs for colorectal cancer were analyzed. They were represented on the blue-coded world map (**Figure 3A**). A total of 40 countries and regions contributed to publications in this area. In terms of research citations, the highest number of citations came from the USA (14290 times), followed by Japan (679 times), Italy (632 times), China (630 times), and the UK (442 times) (**Figure 3B**). Meanwhile, the USA and China had published the fastest growth rates of research in related fields each year in recent years (**Figure 3C**).

A total of 657 institutions published research related to this area. The University of Texas MD Anderson Cancer Center contributed the most publications (23 studies), followed by the Certified Tumor Registrar (15 studies) and the Kaohsiung Medical University (14 studies) (**Figure 3D**). At the same time, The University of Texas MD Anderson Cancer Center and the University of Washington was the institution with the fastest growth rate of published research in recent years (**Figure 3E**).

### Analysis of journals

**Figure 4:**
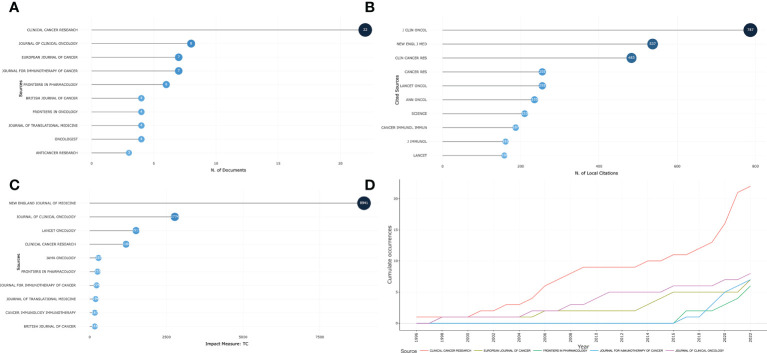
Journals contributing to IRAEs for colorectal cancer. **(A)** Distribution of the top ten journals contributing to the literature. **(B)** Distribution of the top 10 journals with the highest number of citations in published literature. **(C)** The 10 most influential journals in the field. **(D)** Growth curve of the number of journals publishing in the field per year.

A total of 237 articles were published worldwide in 139 journals from 1996 to 2022. Clinical Cancer Research has contributed an enormous amount of research literature (22) (**Figure 4A**). Despite only eight publications, the Journal of Clinical Oncology was the most cited journal, with 787 citations (**Figure 4B**). In the dimension of total citations (TC), the New England Journal of Medicine showed a clean sweep, with 8,941 citations (**Figure 4C**). **Figure 4D** showed the number of journals publishing colorectal cancer immunotherapy side-effect articles per year. As the graph shows, Clinical Cancer Research has been the fastest-growing journal of published research in recent years, followed by the Journal of Clinical Oncology and the European Journal of Cancer.

### Analysis of authors

**Figure 5:**
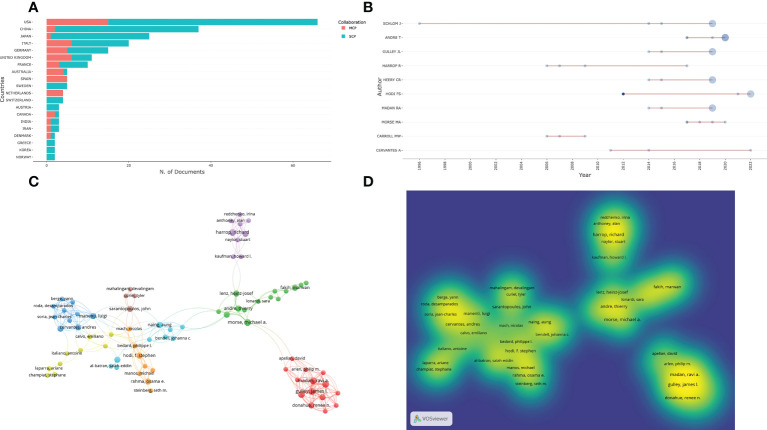
Authors contributing to IRAEs for colorectal cancer. **(A)** Nationality distribution of the contributing authors. **(B)** Distribution of the time of publication of the contributing authors. **(C)** Network map of co-authorship among authors who published more than 5 articles. **(D)** Distribution of co-authors according to the average frequency of occurrence. The keywords in yellow have the highest frequency of occurrence.

Among researchers who published on the IRAEs for colorectal cancer, the USA, China, Japan, Italy, and Germany were the top five countries with the most study authors (**Figure 5A**). Jefferey Schlom has already published one relevant study in 1996, 10 years ahead of the other authors, and had contributed a total of four papers. In addition, Morse MA has contributed one study in a related field in each of the four consecutive years between 2017-2020 (**Figure 5B**). We analyzed 62 authors who had co-authored in more than five publications. After excluding the 12 unlinked items, 40 author collaborations were found (**Figures 5C, D**).

### Citation and co-citation analyses

The citation analysis showed that 17 pieces of literature had more than 100 citations. The top ten most cited publications are listed in **Table 2**. Of these, “Safety, Activity, and Immune Correlates of Anti–PD-1 Antibody in Cancer” (12) has 8431 citations, followed by “Phase I Study of Single-Agent Anti–Programmed Death-1 (MDX-1106) in Refractory Solid Tumors: Safety, Clinical Activity, Pharmacodynamics, and Immunologic Correlates” (13) with 2101 citations. The third most cited article was “Nivolumab in patients with metastatic DNA mismatch repair-deficient or microsatellite instability-high colorectal cancer (CheckMate 142): an open-label, multicenter, phase 2 study” (14) with 1302 citations.

**Table 2:** Information of the 10 most cited documents.

Paper	DOI	Total Citations	TC per Year
TOPALIAN SL, 2012, NEW ENGL J MED	10.1056/NEJMoa1200690	8431	766.45
BRAHMER JR, 2010, J CLIN ONCOL	10.1200/JCO.2009.26.7609	2101	161.62
OVERMAN MJ, 2017, LANCET ONCOL	10.1016/S1470-2045(17)30422-9	1302	217
ANDRE T, 2020, NEW ENGL J MED	10.1056/NEJMoa2017699	510	170
SCOTT AM, 2003, CLIN CANCER RES	Not Applicable	265	13.25
WANG PF, 2017, FRONT PHARMACOL	10.3389/fphar.2017.00730	223	37.17
ENG C, 2019, LANCET ONCOL	10.1016/S1470-2045(19)30027-0	209	52.25
FUKUOKA S, 2020, J CLIN ONCOL	10.1200/JCO.19.03296	202	67.33
SIMONAGGIO A, 2019, JAMA ONCOL	10.1001/jamaoncol.2019.1022	151	37.75
VAN BAREN N, 2005, J CLIN ONCOL	10.1200/JCO.2005.08.375	139	7.72

**Figure 6:**
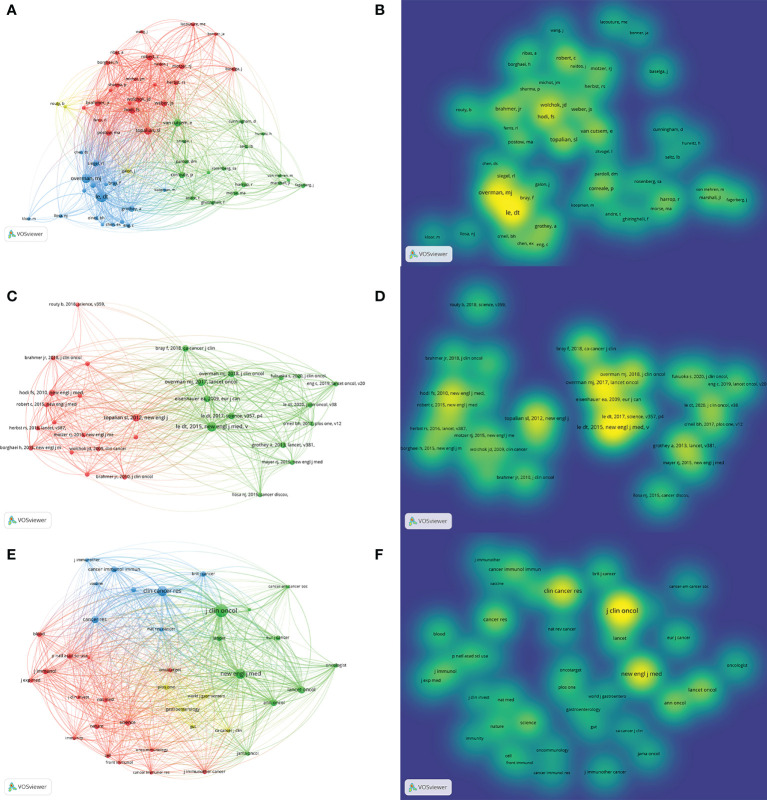
Citation and co-citation analyses of IRAEs for colorectal cancer. **(A)** Network map of authors who have been cited more than 5 times. **(B)** Distribution of authors according to the mean frequency of appearance. **(C)** Network map of references cited more than 10 times **(D)** Distribution of references according to the mean frequency of appearance. **(E)** Network map of documents cited more than 50 times **(F)** Distribution of documents according to the mean frequency of appearance.

In addition, we analyzed 62 authors who co-cited in more than five publications. After excluding the one unlinked item, 61 author collaborations were found (**Figures 6A, B**). The two authors with the highest total link strength were Dung T. Le. (86 total link strengths) and Michael J Overman (58). We also analyzed the co-cited references (**Figures 6C, D**). The five most cited references were “PD-1 Blockade in Tumors with Mismatch-Repair Deficiency” (15) (New England Journal of Medicine; 50 citations), “Nivolumab in patients with metastatic DNA mismatch repair-deficient or microsatellite instability-high colorectal cancer (CheckMate 142): an open-label, multicenter, phase 2 study” (14) (Lancet Oncology; 33 citations), “Safety, activity, and immune correlates of anti-PD-1 antibody in cancer” (12) (New England Journal of Medicine; 29 citations), “Durable clinical benefit with nivolumab plus ipilimumab in DNA mismatch repair-deficient/microsatellite instability-high metastatic colorectal cancer “ (16) (Journal of Clinical Oncology; 25citations) and “Mismatch repair deficiency predicts response of solid tumors to PD-1 blockade” (4) (Science; 24 citations).

**Figures 6E, F** showed the co-cited source journals, 39 of which were cited more than 50 times.

Journal of Clinical Oncology, New England Journal of Medicine, Clinical Cancer Research, Cancer Research, and Lancet Oncology were the most co-cited source journals with 787 co-citations, 537, 483, 255, and 255, respectively.

### Co-occurrence and analysis of author’s keywords and keywords plus

**Figure 7:**
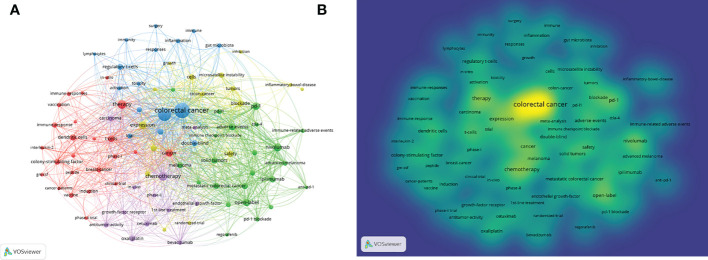
Co-occurrence analysis of keywords. **(A)** Network map of all keywords of studies. **(B)** Distribution of all keywords according to the mean frequency of appearance.

We analyzed all 905 Keywords Plus and 629 Author’s keywords. The density visualization showed the mapping of the exact identified keywords by frequency of occurrence. Our analysis of the author’s keywords and Keywords Plus resulted that co-occurrence>5 times showed that colorectal cancer had the highest link strength of co-occurrence>5 times in all literature at 437, followed by immunotherapy (370), open-label (156), chemotherapy (152), nivolumab (146) and PD-1 (132) (**Figures 7A, B**).

### Trends analysis of all keywords

**Figure 8:**
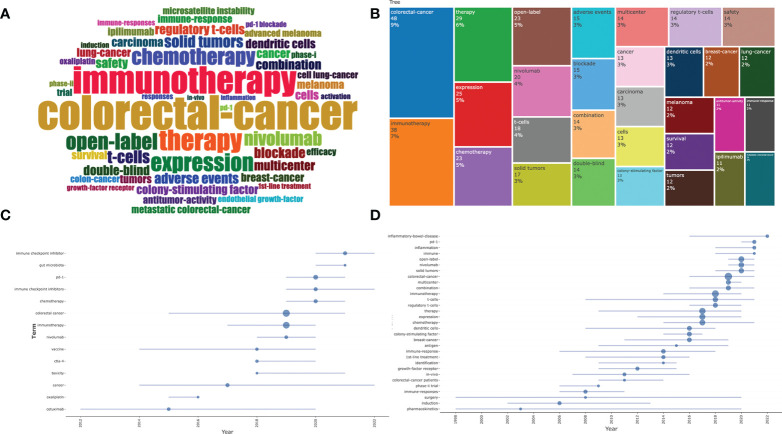
Trend analysis of authors’ keywords and keywords plus. **(A)** Word cloud of authors’ keywords in all literatures. **(B)** Tree map of authors’ keywords. **(C)** Trends in authors’ keywords over time. **(D)** Trends in keywords plus over time.

**Figures 8A, B** shows a cloud map and a tree map of the hot words that emerged from this study. The top five frequencies of occurrence were colorectal cancer, immunotherapy, treatment, expression, and chemotherapy. In addition, we analyzed trends in the author’s keywords and keywords plus over time for all documents separately. As shown in **Figure 8C**, in recent years, the main research hotspots for IRAEs for colorectal cancer include immune checkpoint inhibitors (PD-1, CTLA-4) and gut microbiota. Keywords plus is an expansion of the authors’ keywords, and its distribution could predict the distribution of research hotspots in recent and future years. The results suggested that inflammatory bowel disease may be the key to colorectal cancer-related adverse events in recent years (**Figure 8D**).

## Discussion

Bibliometric analysis methods are rapidly evolving and have been systematically and comprehensively expanded by many researchers (8, 17, 18). The development of research on IRAEs for colorectal cancer is needed based on a large body of clinical data analysis and research reports. Systematic bibliometric analysis is critical in guiding cancer research design and clinical practice (7, 17). We performed the first bibliometric analysis of the research progress and literature on IRAEs in colorectal cancer. In addition, words mining was performed and visualized to extract meaningful information. Words mining could help us to quickly locate keywords in research and hot topics in the progress of this field. In this study, to provide a more accurate landscape of the current state of research on IRAEs for colorectal cancer, we present the results of the bibliometric analysis in visualization, analyzing the role played by countries, institutions, journals, and authors in this emerging field and predicting the hot topics that will continue to be of interest in the coming years. The area had seen a steady increase in the annual publication of relevant journals in the literature since its emergence in 1996. The growth rate has accelerated further in the last few years.

Bibliometric analysis showed that the USA ranks first in the number of publications and citations for IRAEs for colorectal cancer in the analysis of co-authors by country and is currently the world leader in research in this field. It is also at the helm of the direction of research in this area and has significantly impacted the focus of research into colorectal cancer in countries worldwide. The University of Texas MD Anderson Cancer Center made the biggest advancements in the field of study among research institutions. In terms of the quantity of academic articles published, US researcher Dr. Jefferey Schlom had published the most. The citation rate of the literature is a factor that is critical in reflecting its academic impact. Our analysis revealed that literature with high citation rates, broad dissemination, and high impact tends to be randomized controlled trials conducted by internationally renowned medical research institutions.

Notably, among the studies included in this study, the article entitled “Safety, activity, and immune correlates of anti-PD-1 antibody in cancer” by Suzanne L Topalian et al., published in the New England Journal of Medicine in 2012, had a staggering 8,431 citations. This study recruited patients with advanced melanoma, non-small cell lung cancer, prostate cancer, renal cell cancer, or colorectal cancer to receive anti-PD-1 antibodies. To assess its Safety and efficacy. The results showed that the anti-PD-1 antibody produced objective responses in approximately one-quarter to one-fifth of patients with non-small cell lung cancer, melanoma, or renal cell carcinoma, and the safety could be reassuring. Furthermore, “Phase I Study of Single-Agent Anti–Programmed Death-1 (MDX-1106) in Refractory Solid Tumors: Safety, Clinical Activity, Pharmacodynamics, and Immunologic Correlates” and “Nivolumab in patients with metastatic DNA mismatch repair-deficient or microsatellite instability-high colorectal cancer (CheckMate 142): an open-label, multicenter, phase 2 study” had over 1000 cited. A deeper analysis showed that all three of these highly cited papers are randomized controlled trials assessing the efficacy and safety of immunotherapy in the treatment of colorectal cancer as well as other solid tumors. In addition, all three articles were published in some of the world’s most prestigious journals. A co-authorship network involving academics contributing to the current field suggested that close cooperation existed between different research teams, with most researchers involved in multiple research teams. This is due to the fact that collaboration between scholars provided a number of benefits for data collection and research design.

Gut microbiota and inflammatory bowel disease were one of the outstanding results achieved in this study. Our trend analysis concluded that the two topics mentioned above might be one of the hot spots for future research on IRAEs for colorectal cancer. Healthy gut microbiota is essential for developing the immune system and coordinating the immune response. At the same time, dysbiosis is associated with various diseases, including inflammatory bowel disease and colorectal cancer (19, 20). Substantial progress has been made in understanding the molecular pathogenesis of inflammatory bowel disease. Firstly, inflammatory bowel disease is the most complex disease in which susceptibility genes are most easily identified. Furthermore, bacteria (or their products) can act as drivers of immune dysregulation and inflammatory bowel disease rather than traditional pathogens. There is growing evidence that the dynamic balance between microbiota, particularly commensal flora, and the defense of hosts response at the mucosal margin is vital in the initiation and pathogenesis of chronic inflammatory bowel disease (21). Numerous studies have shown an inextricable link between immunity, gut microbiota, and inflammatory bowel disease (21–23). These findings are further supported by our research, which links colorectal cancer, immune-related adverse reactions, gut microbiota, and inflammatory bowel disease, revealing significant directions for future exploration and critical points for research in this area.

A bibliometric analysis will provide valuable references and recommendations, especially for studies of adverse events and side effects of cancer treatments, such as IRAEs in colorectal cancer. It may also provide us with a landscape of literature in the areas of interest to researchers. As a follow-up to this study, we will continue to follow the hot topics and their evolution in the IRAEs in colorectal cancer and plan to conduct updated bibliometric analyses when significant new data become available.

## Conclusions

Our results showed that the USA significantly contributes to the research on IRAEs in colorectal cancer. Most of the studies related to IRAEs in colorectal cancer have been published in high-quality journals, indicating the high attractiveness and impact of research advances in this field. We revealed that the most popular lines of research in this area are inflammatory bowel disease and gut microbiota, indicating that the bibliometric analysis of IRAEs in colorectal cancer has greatly guided future research hotspots as well as directions in colorectal cancer.

## Data availability statement

The original contributions presented in the study are included in the article/supplementary material. Further inquiries can be directed to the corresponding authors.

## Author contributions

JC, YX, and LL contributed to conception and design of the study. LZ organized the database. MS performed the statistical analysis. XH wrote the first draft of the manuscript.XG, LL, YL, and JC wrote sections of the manuscript. All authors contributed to the article and approved the submitted version.

## Conflict of interest

The authors declare that the research was conducted in the absence of any commercial or financial relationships that could be construed as a potential conflict of interest.

## Publisher’s note

All claims expressed in this article are solely those of the authors and do not necessarily represent those of their affiliated organizations, or those of the publisher, the editors and the reviewers. Any product that may be evaluated in this article, or claim that may be made by its manufacturer, is not guaranteed or endorsed by the publisher.
